# Influence of lifestyle and eating behaviours on Mediterranean diet adherence in preschoolers from southern Spain

**DOI:** 10.1017/S1368980025101742

**Published:** 2025-12-26

**Authors:** Gracia Cristina Villodres, Rosario Padial-Ruz, Juan José Pérez-Díaz, José Joaquín Muros

**Affiliations:** 1 Department of Didactics of Corporal Expression, Faculty of Education, University of Granada, Granada, Spain; 2 Department of Physical Education and Sports, Faculty of Sports Sciences, University of Granada, Granada, Spain

**Keywords:** Quality diet, Healthy behaviours, Sedentary behaviours, Childhood

## Abstract

**Objective:**

The present study examined the association of screen and sleep time, physical fitness and eating behaviour with Mediterranean diet (MD) adherence in a sample of preschoolers from Granada, Spain.

**Design:**

A cross-sectional, non-randomised design was employed. A multilinear regression model with backward elimination was used for analysis.

**Setting:**

Variables included age, screen time, hours of nightly sleep, physical fitness, food approach and food avoidance. The developed model met assumptions of multiple regression in terms of linearity, homoscedasticity, normality, independence and non-multicollinearity.

**Participants:**

Data were collected from 653 of the 2250 three-to-six-year-old children attending the eighteen schools invited to take part in the present study. Data on children’s behaviours were parent-reported using validated questionnaires.

**Results:**

Better sleep time and lower screen time and food avoidance were found to be predictive of MD adherence. These variables explained 15 % of the variance in preschoolers’ MD adherence.

**Conclusions:**

The present study suggests that sleep and screen time and food avoidance are important components to consider when targeting improvements in MD adherence in preschoolers. Future research should explore the way in which parental health behaviours influence their children’s health habits in order to better understand the outcomes.

To encourage healthy growth, children under 6 years old should spend limited time sitting in front of screens or being restrained in strollers and seats. New guidelines from the WHO urge better sleep habits and increased opportunities for active play^(1)^. In relation to this, the WHO reported in 2022 that 37 million children under 6 years of age were overweight or obese^(2)^. Specifically, Spain is amongst a number of European countries with rates of approximately 25 % of preschool children being overweight or obese^(3)^.

Childhood obesity is primarily caused by an imbalance in which energy intake exceeds energy expenditure, with a genetic predisposition towards weight gain also being a major factor^(4)^. However, a singular genetic or endocrine factor does not explain weight gain in the majority of obese children^(5)^. This indicates that lifestyle factors are the primary contributors to the early onset of obesity.

In this context, a study by Morais-López *et al*.^(6)^ indicates that preschool children from Mediterranean countries within the European Union who do not closely follow a Mediterranean diet (MD) are more likely to be overweight or obese. The MD is characterised by a low saturated fat content and high intake of plant-based foods such as fruits, vegetables, whole grains, nuts and seeds. Olive oil is the primary source of fat, and it comprises moderate amounts of dairy products such as cheese and yogurt, fish and poultry, with red meat consumption being limited^(7)^. Additionally, the MD is characterised by cooking methods and a lifestyle that promotes physical activity, adequate rest and social interaction during meals^(8)^. Specifically in the preschool population, adequate MD adherence plays a protective role against health issues that are highly common at this stage, such as wheezing and acute rhinosinusitis^(9,10)^. Furthermore, adequate MD adherence is associated with better physical fitness in later years^(11)^, with physical fitness being a protective factor against obesity in Spanish children^(12)^.

Despite all of the health benefits of MD adherence, preschool children are continuously exposed to sugary drinks and snacks. Increased consumption of energy-dense food and beverages, along with decreased fruit and vegetable intake, has been shown to be associated with greater screen time^(13)^. Spending time in front of screens is a harmful sedentary habit, especially because it has been shown to lead to the unconscious consumption of high-energy foods and soft drinks in European preschool children^(14)^. Likewise, less healthy eating habits are linked with shorter sleep duration^(15)^.

According to Todendi *et al*.^(16)^, alongside the aforementioned factors related to eating habits, food preferences also play a significant role at this stage. Eating behaviour is established during the early years of childhood, although it can change over time as a function of lived experiences^(17)^. Such experiences pertain to a wide range of activities and decisions related to the selection, preparation and consumption of foods, which are influenced by various biological, psychological, social and cultural factors^(18)^. With regard to that discussed above, poorer eating habits such as ‘mindless eating’ or the inclination towards ‘reward foods’ (often high in sugar) are associated with increased screen time and shorter sleep duration^(19,20)^.

Further, Di Nucci *et al*.^(21)^ observed in a sample of 3- to 11-year-old Italian children that those with lower food neophobia reported higher intakes of fruits, vegetables and legumes. In contrast, children with higher food neophobia reported lower fish consumption and more frequently consumed fast food. Thus, high MD adherence may be a protective factor when it comes to eating behaviour, specifically with regard to the control of emotional eating^(22)^ and food avoidance^(21)^. Establishing such habits early ensures better health in adulthood^(17)^.

Perusal of existing literature reveals a scarcity of scientific output regarding MD adherence and eating behaviour in Spanish preschoolers aged between 3 and 6 years old.

In order to gain a better understanding of MD adherence and eating behaviour during childhood, it is important to explore potential determinants. Thus, the aim of the present study was to examine the association of screen and sleep time, physical fitness and eating behaviour with MD adherence in a sample of preschoolers from Granada, Spain.

## Material and methods

A total of 2250 children were invited to participate in the study. Of these, 659 children were initially recruited following receipt of parental consent and completed questionnaires. Six children were excluded due to incomplete data, resulting in a final sample of 653 participants (336 girls and 317 boys). Full details pertaining to recruitment have been previously reported^(23)^. Some minor modifications were made to the method described in that study, with the same instruments being applied but focusing on different aspects of the measures. These changes are explained in the following sections.

### Instruments

As participants were preschool-aged children, all instruments were administered as parent-reported questionnaires.

MD adherence was evaluated using the latest updated version of the Mediterranean Diet Quality Index (KIDMED)^(24)^. This questionnaire consists of sixteen dichotomous items related to MD patterns. Twelve of the items are positively framed with positive responses attributed a score of +1, whilst four of the items are negatively framed with positive responses attributed a score of –1. Scores for the sixteen items were then summed to produce a total score. Based on this score, MD adherence was classified as high (≥8), medium (4–7) and low (≤3). Medium and low MD adherence were grouped together due to the low percentage of children reporting low MD adherence (3·8 %).

Parental perceptions of children’s eating behaviours were evaluated using a version of the Child Eating Behaviour Questionnaire (CEBQ) developed by Wardle *et al*.^(25)^, which had been previously translated and validated in Spanish^(26)^. This questionnaire consists of thirty-five items, which are rated along a five-point Likert scale. Items are divided into eight subscales. Four subscales pertain to interest in food (food responsiveness, emotional overeating, enjoyment of food, desire to drink), whilst the other four subscales pertain to a lack of interest in food (satiety responsiveness, slowness in eating, emotional undereating, food fussiness). Definitions of these concepts are presented in Table 1, and the full questionnaire is provided in the Supplementary Material. The scores of items belonging to the same subscale were summed, and the means and standard deviations were calculated.

**Table 1.** Definitions of Child Eating Behaviour Questionnaire concepts

**Food approach**	
Food responsiveness	Susceptibility to prefer foods with better organoleptic properties in everyday contexts.
Emotional overeating	Tendency to increase intake in negative emotional contexts.
Enjoyment of food	Condition positively associated with feelings of hunger, desire to eat and pleasure from food.
Desire to drink	Desire to drink and tendency to drink liquids.
**Food avoidance**	
Satiety responsiveness	Decrease in the feeling of hunger caused by food consumption.
Slowness in eating	Tendency to eat more slowly over the course of a meal and to prolong its duration.
Emotional undereating	Tendency to reduce intake in negative emotional contexts.
Food fussiness	Conditional requirement that limits the range of food products that are accepted.

The latest Spanish version of the International Fitness Scale^(27)^ was used to evaluate physical fitness. This questionnaire consists of five items, with responses being provided along a five-point scale (very poor = 1; poor = 2; fair = 3; good = 4; very good = 5). Four items address individual core dimensions of self-perceived physical fitness (cardiorespiratory fitness, muscular fitness and balance), and one item addresses overall physical fitness. The five items are then summed to produce a total score. The full questionnaire is available in the Supplementary Material.

An *ad hoc* questionnaire was designed to collect socio-demographic data. Parents reported their children’s sex, data of birth, height and weight. Parents also reported the number of hours their child slept at night and their child’s daily screen time on weekdays and at weekends. An overall summary score was calculated as the mean number of hours reported over the 7 d examined. Furthermore, parents reported their child’s height and weight. To do this, they were asked to measure and weigh their children at a pharmacy within a determined time period. BMI was then calculated from height and weight (BMI = weight [kg]/(height [m])2). The complete questionnaire is available in the Supplementary Material.

### Statistical analysis

Means and standard deviations were reported for all quantitative variables. Normality of the data was tested using the Kolmogorov–Smirnov test. After verifying that the variables were not normally distributed, data were analysed according to the Mann–Whitney *U* test. Effect sizes were calculated according to Cohen’s *d* and interpreted as small (*d* = 0·2), moderate (*d* = 0·5) or large (*d* = 0·8)^(28)^. Associations between quantitative variables were examined according to Spearman correlations. Finally, a multiple regression model was developed using forward elimination. The aim of this was to identify predictors of MD adherence and control for confounding variables. This analysis considered the variables of age, screen time, hours of nightly sleep, physical fitness, food approach and food avoidance. This analysis was then repeated as a function of sex. Assumptions of linearity, homoscedasticity and normality were verified prior to analysis. All statistical analyses were performed using version twenty-five of IBM-SPSS® for Windows. Statistical significance was established at 0·05.

## Results

Descriptive characteristics pertaining to the study sample are presented in Table 2 as a function of MD adherence and sex. Analysis according to MD adherence revealed significant differences regarding screen time and sleep time. Children with higher MD adherence reported lower screen time (1·43 (sd 0·92) *v*. 1·91 (sd 1·09) h; *P* ˂ .001; *d* = .476) and greater sleep time (10·46 (sd 0·58) *v*. 10·32 (sd 0·68) h; *P* = .008; *d* = .222) than children with low-to-moderate MD adherence. No significant differences were found with regard to overall physical fitness. However, children with high MD adherence exhibited greater physical fitness (4·39 (sd 0·59) *v*. 4·24 (sd 0·66); *P* = .018; *d* = .240) and balance (4·18 (sd 0·71) *v*. 4·04 (sd 0·71); *P* = .018; *d* = .197) than children with low-to-moderate MD adherence. With regard to CEBQ outcomes, no significant differences were found in relation to food approach. However, children with low-to-moderate MD adherence scored higher for emotional overeating (1·78 (sd 0·63) *v*. 1·70 (sd 0·69); *P* = .028; *d* = .121) and lower for enjoyment of food (3·50 (sd 0·81) *v*. 3·85 (sd 0·69); *P* ˂ .001; *d* = .530) than children with high MD adherence. Statistically significant differences were found regarding food avoidance, with higher scores in children with low-to-moderate MD adherence than in children with high MD adherence (2·97 (sd 0·57) *v*. 2·72 (sd 0·45); *P* < .001; *d* = .486). Higher scores were reported for all subdimensions by children with low-to-moderate MD adherence. Analysis according to sex revealed significant differences regarding sleep time. Specifically, boys reported less sleep time than girls (10·29 (sd 0·61) *v*. 10·41 (sd 0·70); *P* = .011; *d* = .183). No significant differences were found for age, BMI, screen time, MD adherence, overall physical fitness or food approach. However, girls reported higher scores for food avoidance than boys (2·95 (sd 0·53) *v*. 2·86 (sd 0·57); *p* = .019; *d* = .164), especially with regard to satiety responsiveness (2·88 (sd 0·64) *v*. 2·75 (sd 0·69); *p* = .004; *d* = .210).

**Table 2.** Descriptive characteristics of the study sample according to MD adherence and sex

	Overall (*n* 653)		High (*n* 173)		Low-mod (*n* 480)		*P*	Cohen’s *d*	Male (*n* 317)		Female (*n* 336)		*P*	Cohen’s *d*
	Mean	sd	Mean	sd	Mean	sd			Mean	sd	Mean	sd		
Age (years)	4·78	0·93	4·80	0·89	4·77	0·95	.642	.033	4·78	0·97	4·78	0·90	.892	.000
BMI (kg/m^2^)	15·53	2·09	15·47	1·99	15·55	2·12	.792	.039	15·52	1·92	15·53	2·23	.691	.005
Screen time (hours)	1·78	1·07	1·43	0·92	1·91	1·09	<.001	.476	1·81	1·11	1·75	1·02	.604	.056
Sleep time (hours)	10·35	0·66	10·46	0·58	10·32	0·68	.008	.222	10·29	0·61	10·41	0·70	.011	.183
MD adherence									7·18	2·09	7·16	1·97	.999	.010
Total PF	4·11	0·65	4·18	0·53	4·08	0·58	.092	.180	4·14	0·59	4·08	0·55	.104	.105
Overall PF	4·28	0·65	4·39	0·59	4·24	0·66	.018	.240	4·30	0·67	4·26	0·62	.267	.062
CF	4·05	0·70	4·11	0·70	4·03	0·76	.335	.109	4·09	0·76	4·02	0·74	.203	.093
MS	4·02	0·70	4·05	0·70	4·02	0·70	.600	.043	4·06	0·71	3·99	0·69	.223	.099
SA	4·12	0·72	4·20	0·68	4·10	0·74	.142	.141	4·17	0·72	4·07	0·69	.074	.142
Balance	4·07	0·71	4·18	0·71	4·04	0·71	.018	.197	4·08	0·74	4·07	0·69	.765	.014
Food approach	2·50	0·56	2·53	0·52	2·49	0·57	.344	.073	2·50	0·56	2·51	0·56	.998	.018
FR	2·22	0·84	2·22	0·83	2·22	0·84	.910	.000	2·17	0·80	2·27	0·87	.258	.120
EO	1·76	0·64	1·70	0·69	1·78	0·63	.028	.121	1·74	0·65	1·77	0·64	.442	.047
EF	3·59	0·80	3·85	0·69	3·50	0·81	<.001	.530	3·60	0·82	3·58	0·78	.407	.025
DD	2·46	0·87	2·36	0·76	2·49	0·91	.315	.155	2·48	0·91	2·43	0·84	.560	.057
Food avoidance	2·91	0·55	2·72	0·45	2·97	0·57	<.001	.486	2·86	0·57	2·95	0·53	.019	.164
SR	2·81	0·67	2·64	0·60	2·89	0·68	<.001	.390	2·75	0·69	2·88	0·64	.004	.210
SE	3·08	0·79	2·96	0·73	3·12	0·80	.040	.209	3·02	0·81	3·13	0·77	.050	.139
EU	2·96	0·60	2·90	0·52	2·99	0·62	.064	.157	2·94	0·60	2·98	0·60	.429	.067
FF	2·76	0·84	2·37	0·68	2·90	0·84	<.001	.694	2·77	0·86	2·80	0·81	.193	.036

MD, Mediterranean diet; PF, physical fitness; CF, cardiorespiratory fitness; MS, muscular strength; B, balance; FR, food responsiveness; EO, emotional overeating; EF, enjoyment of food; DD, desire to drink; SR, satiety responsiveness; SE, slowness in eating; EU, emotional undereating; FF, food fussiness. MD adherence: high (≥8 points), low-mod (≤7 points; medium [4–7] and low [≤3] combined due to the small number of participants with low adherence). Overall: results for the entire sample.

Table 3 presents correlation coefficients pertaining to the relation of MD adherence with age, screen time, sleep time, overall physical fitness, food approach and food avoidance. Higher MD adherence was positively associated with sleep time (*r* = .212; *P* **<** .001) and overall physical fitness (*r* = .092; *P* **<** .05). However, the latter association only emerged in males (*r* = .117; *P* **<** .05). Negative associations were found with screen time (*r* = −.257; *P* **<** .001) and food avoidance (*r* = −.240; *P* **<** .001) in both females and males.

**Table 3.** Correlation coefficients pertaining to the relationship of MD adherence with age, screen time, sleep time, overall physical fitness, food approach and food avoidance

MD adherence	Age	Screen time	Sleep time	Total PF	Food approach	Food avoidance
Total	.009	−.257**	.212**	.092*	.035	−.240**
Girls	.027	−.284**	.196**	.070	.041	−.203**
Boys	−.007	−.228**	.226**	.117*	.029	−.273**

*P < 0·05; **P < 0·01. MD, Mediterranean diet; PF, physical fitness.

Given the high number of variables involved, a stepwise procedure was employed in order to consider the influence of potential confounding variables on the association of interest (Table 4). Lower screen time, lower food avoidance and a greater number of hours of nightly sleep were found to be predictive of MD adherence. These variables explained 15 % of the variance detected in MD adherence. When considering sex-related differences, lower food avoidance, greater number of hours of nightly sleep and lower screen time emerged as predictors, explaining 15·2 % of variance in MD adherence in girls and 16 % of variance in boys.

**Table 4.** Predictors of Mediterranean diet adherence

	Beta	*t*	*p*	95 % CI	*R*2
Total					.150
Screen time	−.196	− 5·238	˂.001	[−.510, −.232]	
Food avoidance	−.222	− 6·074	˂.001	[−1·085, −.555]	
Sleep time	.172	4·619	˂.001	[.304, .755]	
Boys					.160
Food avoidance	−.286	− 5·443	˂.001	[−1·435, −.673]	
Sleep time	.169	3·176	.002	[.221, .942]	
Screen time	−.139	− 2·573	.011	[−.460, −.061]	
Girls					.152
Screen time	−.250	− 4·819	˂.001	[−.675, −.284]	
Sleep time	.180	3·466	.001	[.220, .798]	
Food avoidance	−.156	3·054	.002	[−.955, −.207]	

## Discussion

The main finding of the present study is that screen time, food avoidance and sleep time are reasonably good predictors of MD adherence in this group of children (together accounting for 15 % of variance).

The present findings suggest that screen time and sleep time are good predictors of MD adherence. Sørensen *et al*. reported similar outcomes in a sample of 3- to 7-year-old Norwegian children, finding both screen and sleep time to be predictors of diet quality. In this case, screen time emerged as the strongest predictor^(29)^. This same study reported that screen and sleep time at both 18 months and 3 years old were prospectively associated with diet quality. Generally, most evidence links screen time, acknowledged as a primary sedentary behaviour, with poor diet quality in children^(30)^. Further, another study tracking sleep patterns from 6 months of age up until 7 years of age found that shorter sleep duration in early life was linked to poorer diet quality later on^(31)^.

In line with the present study, preschoolers with higher MD adherence exhibited better overall physical fitness, higher sleep time and lower time spent in front of screens. Other studies have reported similar outcomes in 4- to 5-year-old Spanish preschoolers^(32)^. In this case, participants with higher MD adherence also met recommendations for physical activity (180 min of which 60 min should involve vigorous play), screen time (no more than 60 min a day) and sleep time (10–13 h of high-quality sleep). One possible explanation for this relationship is that dietary habits that are more aligned with the MD may be associated with improved physical fitness, reduced sedentary behaviour and better overall health between 3 and 18 years of age^(33)^.

In addition to screen and sleep time, food avoidance was a significant predictor of MD adherence in the present study. In this sense, children with lower MD adherence reported greater food avoidance. Food avoidance and food neophobia are related concepts as they both involve the avoidance of certain foods. Anjos *et al*.^(34)^ observed that 3- to 6-year-old children with higher food neophobia frequently consumed ultra-processed foods rich in sugars (such as snacks, cookies and sweets), which are associated with unhealthy eating behaviours and poorer diet quality. At the same time, similar outcomes have been observed in primary (8–11 years) and secondary (12–18 years) school children, with lower MD adherence being exhibited by neophobic schoolchildren. In this regard, according to Di Nucci, food rejection can steer children away from following an MD^(21)^.

With regard to food approach, children with lower MD adherence reported higher emotional overeating and lower enjoyment of food in the present study. Buja *et al*. reported similar outcomes in 8- to 9-year-old children, with optimal MD adherence being associated with a lower risk of emotional overeating^(22)^. In 5- to 12-year-old children, a relationship has been observed between emotional overeating and stressful events and negative emotions. Specifically, children with greater emotional problems report higher sweet and fatty food consumption and more unhealthy dietary patterns in general^(35)^. Typically, when individuals experience negative emotional symptoms, they tend to seek out sweet and palatable foods to improve their mood^(36)^. Thus, parents of preschool children often offer unhealthy snacks as an emotional regulation strategy, leading the child to be prone to overeating and vulnerable to developing a link between food and pleasure. This may lead to a dependency on food as a means of managing emotions, rather than consuming it to meet their nutritional requirements^(37)^. In another sense, in order to understand why higher food enjoyment is associated with high MD adherence, it must be borne in mind that following an MD involves social interactions during mealtimes^(8)^. In this sense, Torre-Moral *et al*.^(38)^ reported that MD adherence relates to the manner in which people eat and the enjoyment derived from sharing meals with important individuals. In this sense, a highly researched concept pertains to ‘family meals’, which involves the pleasure of eating together with family.

With regard to sex differences, although girls reported greater food avoidance than boys, in the present study, food avoidance was a stronger predictor of MD adherence in boys. Most evidence suggests that males are generally more neophobic than females^(39)^, with males tending to consume more soft drinks, red meat and pasta and less fruits and vegetables than women of the same age^(39)^. Similarly, women often show greater enthusiasm for health and natural products and tend to be more committed to eating nutritious foods^(40)^, which includes engaging in more positive eating behaviours as shown through the higher satiety responsiveness scores observed in the present study. In this sense, girls in particular may be more likely to imitate the eating behaviours of their mothers, since other studies show that maternal education on feeding directly influences factors affecting eating in young children^(41)^.

Additionally, screen time is a stronger predictor of MD adherence in girls. Females are generally more sedentary than males, partly due to access barriers that result in lower physical activity engagement^(42)^, with screen use accounting for large amounts of sedentary behaviour in Spain. Thus, differences in these behaviours amongst females may influence the adoption of healthy or unhealthy habits. Against this backdrop, whilst 3- to 6-year-old children may not fully understand these concepts, such behaviours can be culturally learned from their parents and influence their own habits.

The present findings should also be interpreted considering the strong influence of parental behaviours on children’s health habits. Parents act as primary role models, shaping children’s dietary preferences, screen exposure and sleep routines through their own habits and household practices. Previous research has shown that parental feeding practices and attitudes toward food are directly associated with children’s diet quality and emotional eating^(43,44)^. Therefore, family-based interventions that actively engage parents may be more effective in improving MD adherence during early childhood. According to Gray *et al*.^(44)^, additional research is needed to better understand these parental influences, which could help enhance the effectiveness of nutrition interventions aimed at changing children’s dietary behaviours.

## Limitations of this study

The conclusions drawn from the present study should be considered in light of several limitations. One major limitation is the cross-sectional nature of the research, which restricts its ability to explore causal relationships. In particular, the cross-sectional design does not allow us to determine the direction of the associations. For example, better sleep patterns might lead to improved dietary habits by reducing fatigue and emotional eating, but conversely, adherence to a healthy dietary pattern could promote better sleep quality. This bidirectional relationship is especially relevant in young children, where behavioural and physiological mechanisms are still developing, and highlights the need for longitudinal studies to clarify the temporal order of these effects. Additionally, reliance on self-report data for several variables increases the risk of measurement error. However, given that both the KIDMED for children and the CEBQ have shown high validity and reliability in similar populations, this limitation is unlikely to have had a meaningful impact on study findings. Another limitation is that the sample was not randomly selected. Despite this, a sufficiently large number of participants were recruited from eighteen public and private schools in the province of Granada. A further limitation is the lack of a direct measure of socio-economic status, as parents of participating preschoolers did not consent to its collection. Socio-economic disadvantage may influence both diet and sleep quality, meaning that some of the observed associations could be confounded by deprivation. Although other potential confounders were controlled for, the independence of these associations from socio-economic factors cannot be fully established.

However, it must be acknowledged that the present study is, to the authors’ knowledge, one of the few studies to analyse the association of MD adherence with screen and sleep time, physical fitness and eating behaviour in a sample of children under 6 years of age.

## Conclusions

The present study suggests that screen time, sleep time and food avoidance are important factors to consider when targeting improvements in MD adherence in 3- to 6-year-old children. Preschoolers with optimal MD adherence reported more sleep time and better overall physical fitness than children with poor MD adherence. In contrast, the latter group reported spending more time in front of screens and exhibited greater food avoidance. Thus, it is concluded that interventions aimed at tackling food avoidance, reducing screen time and increasing sleep time are needed to promote MD adherence in preschoolers.

From a practical perspective, these findings support the need for family- and school-based health promotion programmes aimed at reducing screen exposure, improving sleep routines and fostering positive eating behaviours from early childhood. Policymakers and educators should involve parents as active agents in interventions promoting MD patterns amongst preschoolers. At the same time, it would be valuable to conduct a study examining the influence of parents’ healthy habits on their children’s behaviours, as this could provide further insight into the results produced by the present study.

## Supporting information

Villodres et al. supplementary material Villodres et al. supplementary material

## Supplementary material

For supplementary material accompanying this paper visit https://doi.org/10.1017/S1368980025101742
