# Supplementary material for: Influence of lifestyle and eating behaviours on Mediterranean diet adherence in preschoolers from southern Spain
Source: Public Health Nutr. 2025 Dec 26;29(1):e56. doi: 10.1017/S1368980025101742 (PMC13112296; doi:10.1017/S1368980025101742)
Supplement: Villodres et al. supplementary material [file S1368980025101742sup001.docx]

**Supplementary Material – Instruments (Spanish version)**

| **DATOS SOCIODEMOGRÁFICOS DE SU HIJO/A** | | | | | | | | | | | | | |
| --- | --- | --- | --- | --- | --- | --- | --- | --- | --- | --- | --- | --- | --- |
| **Sexo** | | | Hombre | | | | | | Mujer | | | | |
| **Talla (en metros)** | | |  | | | | | | **Peso (en kg)** | |  | | |
| **Fecha de medición de talla y peso** | | |  | | | | | | | | | | |
| **Fecha de nacimiento del niño/a** | | |  | | | | | | | | | | |
| **TEST DE AUTOEVALUACIÓN DE CONDICIÓN FÍSICA DE SU HIJO/A (IFIS)** | | | | | | | | | | | | | |
| **1. Mi condición física general es:** | | | | | | | | | | | | | |
| Muy mala | | Mala | | | Aceptable | | Buena | | | | | Muy buena | |
| **2. Mi condición física cardio-respiratoria (capacidad para hacer ejercicio, por ejemplo, correr durante mucho tiempo) es:** | | | | | | | | | | | | | |
| Muy mala | | Mala | | | Aceptable | | Buena | | | | | Muy buena | |
| **3. Mi fuerza muscular es:** | | | | | | | | | | | | | |
| Muy mala | | Mala | | | Aceptable | | Buena | | | | | Muy buena | |
| **4. Mi velocidad/agilidad es:** | | | | | | | | | | | | | |
| Muy mala | | Mala | | | Aceptable | | Buena | | | | | Muy buena | |
| **5. Mi equilibrio es:** | | | | | | | | | | | | | |
| Muy mala | | Mala | | | Aceptable | | Buena | | | | | Muy buena | |
| **TEST DE HÁBITOS SEDENTARIOS Y DE SUEÑO DE SU HIJO/A** | | | | | | | | | | | | | |
| **1.** ¿Cuántas horas diarias dedica su hijo/a a actividades de ocio (frente a una pantalla) en **DÍA DE SEMANA**? Por ejemplo: Ver la televisión, jugar a videojuegos, usar el móvil, usar el ordenador, etc. (No tenga en cuenta las horas de clases online, solo su uso en su tiempo libre) | | | | | | | | H | | | | MIN | |
| **2.** ¿Cuántas horas diarias dedica su hijo/a a actividades de ocio (frente a una pantalla) en **DÍAS DE FIN DE SEMANA**? Por ejemplo: Ver la televisión, jugar a videojuegos, usar el móvil, usar el ordenador, etc. (No tenga en cuenta las horas de clases online, solo su uso en su tiempo libre) | | | | | | | | H | | | | MIN | |
| **3.** ¿A qué hora se suele acostar su hijo/a los **DÍAS DE SEMANA**? | | | | | | | | H | | | | MIN | |
| **4.** ¿A qué hora se suele acostar su hijo/a los **DÍAS DE FIN DE SEMANA**? | | | | | | | | H | | | | MIN | |
| **5.** ¿A qué hora se suele levantar su hijo/a los **DÍAS DE SEMANA**? | | | | | | | | H | | | | MIN | |
| **6.** ¿A qué hora se suele levantar su hijo/a los **DÍAS DE FIN DE SEMANA**? | | | | | | | | H | | | | MIN | |
| **TEST DE ADHERENCIA A LA DIETA MEDITERRÁNEA DE SU HIJO/A (KIDMED)** | | | | | | | | | | | | | |
| **(MARCA SIEMPRE CON UNA X)** | | | | | | | | | | | **SÍ** | | **NO** |
| **1. Toma una fruta todos los días.** | | | | | | | | | | |  | |  |
| **2. Toma una 2ª pieza de fruta todos los días.** | | | | | | | | | | |  | |  |
| **3. Toma verduras frescas (ensaladas) o cocinadas regularmente una vez al día.** | | | | | | | | | | |  | |  |
| **4. Toma verduras frescas o cocinadas de forma regular más de una vez al día.** | | | | | | | | | | |  | |  |
| **5. Consume pescado con regularidad (por lo menos 2-3 veces al a semana).** | | | | | | | | | | |  | |  |
| **6. Acude una vez o más a la semana a un centro de comida rápida (fast food) tipo hamburguesería.** | | | | | | | | | | |  | |  |
| **7. Le gustan las legumbres y las toma más de 1 vez a la semana.** | | | | | | | | | | |  | |  |
| **8. Toma pasta integral o arroz integral casi a diario (5 días o más a la semana).** | | | | | | | | | | |  | |  |
| **9. Desayuna un cereal integral o derivado integral (pan integral, etc.).** | | | | | | | | | |  | | |  |
| **10. Toma frutos secos con regularidad (al menos 2-3 veces a la semana).** | | | | | | | | | |  | | |  |
| **11. Se utiliza aceite de oliva en casa.** | | | | | | | | | |  | | |  |
| **12. Se salta el desayuno.** | | | | | | | | | |  | | |  |
| **13. Desayuna un lácteo (yogurt, leche, etc.).** | | | | | | | | | |  | | |  |
| **14. Desayuna bollería industrial, galletas o pastelitos.** | | | | | | | | | |  | | |  |
| **15. Toma 2 yogures y/o 40 g queso cada día.** | | | | | | | | | |  | | |  |
| **16. Toma golosinas y/o caramelos varias veces al día.** | | | | | | | | | |  | | |  |
| **TEST DE COMPORTAMIENTO ALIMENTARIO DE SU HIJO/A (CEBQ)** | | | | | | | | | | | | | |
| **1. A mi hijo/a le encanta la comida**. | | | | | | | | | | | | | |
| Nunca | Casi nunca | | | Algunas veces | | A menudo | | | | Siempre | | | |
| **2. Mi hijo/a come más cuando está preocupado.** | | | | | | | | | | | | | |
| Nunca | Casi nunca | | | Algunas veces | | A menudo | | | | Siempre | | | |
| **3. Mi hijo/a tiene un gran apetito.** | | | | | | | | | | | | | |
| Nunca | Casi nunca | | | Algunas veces | | A menudo | | | | Siempre | | | |
| **4. Mi hijo/a termina su comida muy rápido.** | | | | | | | | | | | | | |
| Nunca | Casi nunca | | | Algunas veces | | A menudo | | | | Siempre | | | |
| **5. Mi hijo/a tiene interés en las comidas y los alimentos.** | | | | | | | | | | | | | |
| Nunca | Casi nunca | | | Algunas veces | | A menudo | | | | Siempre | | | |
| **6. Mi hijo/a siempre está pidiendo algo de beber.** | | | | | | | | | | | | | |
| Nunca | Casi nunca | | | Algunas veces | | A menudo | | | | Siempre | | | |
| **7. Mi hijo/a rechaza los alimentos que no conoce cuando se le ofrecen por primera vez.** | | | | | | | | | | | | | |
| Nunca | Casi nunca | | | Algunas veces | | A menudo | | | | Siempre | | | |
| **8. Mi hijo/a come despacio.** | | | | | | | | | | | | | |
| Nunca | Casi nunca | | | Algunas veces | | A menudo | | | | Siempre | | | |
| **9. Mi hijo/a come menos cuando está enfadado.** | | | | | | | | | | | | | |
| Nunca | Casi nunca | | | Algunas veces | | A menudo | | | | Siempre | | | |
| **10. Mi hijo/a disfruta probando nuevos alimentos.** | | | | | | | | | | | | | |
| Nunca | Casi nunca | | | Algunas veces | | A menudo | | | | Siempre | | | |
| **11. Mi hijo/a come menos cuando está cansado.** | | | | | | | | | | | | | |
| Nunca | Casi nunca | | | Algunas veces | | A menudo | | | | Siempre | | | |
| **12. Mi hijo/a siempre está pidiendo comida.** | | | | | | | | | | | | | |
| Nunca | Casi nunca | | | Algunas veces | | A menudo | | | | Siempre | | | |
| **13. Mi hijo/a come más cuando está molesto o irritado.** | | | | | | | | | | | | | |
| Nunca | Casi nunca | | | Algunas veces | | A menudo | | | | Siempre | | | |
| **14. Si se le permitiera, mi hijo/a comería demasiado.** | | | | | | | | | | | | | |
| Nunca | Casi nunca | | | Algunas veces | | A menudo | | | | Siempre | | | |
| **15. Mi hijo/a come más cuando está nervioso o inquieto.** | | | | | | | | | | | | | |
| Nunca | Casi nunca | | | Algunas veces | | A menudo | | | | Siempre | | | |
| **16. Mi hijo/a disfruta de una gran variedad de alimentos.** | | | | | | | | | | | | | |
| Nunca | Casi nunca | | | Algunas veces | | A menudo | | | | Siempre | | | |
| **17. Mi hijo/a deja comida en el plato al final de la comida.** | | | | | | | | | | | | | |
| Nunca | Casi nunca | | | Algunas veces | | A menudo | | | | Siempre | | | |
